# Factors Associated with Nursing Professionalism: Insights from Tertiary Care Center in India

**DOI:** 10.1186/s12912-024-01820-4

**Published:** 2024-03-06

**Authors:** Poonam Kumari, Surya Kant Tiwari, Nidhin Vasu, Poonam Joshi, Manisha Mehra

**Affiliations:** 1 Department of Nursing Services, All India Institute of Medical Sciences, Kalyani, West Bengal India; 2 College of Nursing, All India Institute of Medical Sciences, Raebareli, Uttar Pradesh India; 3 College of Nursing, All India Institute of Medical Sciences, Kalyani, West Bengal India; 4 College of Nursing, All India Institute of Medical Sciences, Patna, Bihar India

**Keywords:** India, Patient safety, Policy, Professionalism, Regression analysis, Tertiary care center, Cross-sectional studies

## Abstract

**Background:**

Professionalism among nurses plays a critical role in ensuring patient safety and quality care and involves delivering competent, safe, and ethical care while also working with clients, families, communities, and healthcare teams.

**Aims and objectives:**

To assess the level of nursing professionalism and the factors affecting professionalism among nurses working at a tertiary care center in India.

**Methods:**

A descriptive cross-sectional study was conducted from October 2022 to March 2023 using a total enumeration sampling technique. Following institutional ethics committee approval, standardized tools were administered consisting of Nursing Professionalism Scale and socio-demographic, personal, and organizational characteristics.

**Results:**

A total of 270 nurses participated, with a response rate of 93.7%. The mean age of the participants was 27.33 ± 2.75 years, with the majority being female (82.6%) and belonged to the age group of 23–27 years (59.6%). More than half of the nurses exhibited high professionalism (53%), with the highest and lowest median scores for professional responsibility (29.0) and valuing human beings (13.0) respectively. Multivariate regression analysis demonstrated that, compared with their counterparts, nurses with a graduate nursing qualification (AOR = 4.77, 95% CI = 1.16–19.68), up-to-date training (AOR = 4.13, 95% CI = 1.88–9.06), and adequate career opportunity (AOR = 33.91, 95% CI = 14.48–79.39) had significant associations with high nursing professionalism.

**Conclusion/Implications for practice:**

The majority of the nurses had high professionalism, particularly in the domains of professional responsibility and management. Hospitals and healthcare institutions can use these findings to develop policies and prioritize opportunities for nurses to attend conferences and workshops to enhance their professional values, ultimately leading to improved patient care outcomes.

**Patient and public contribution:**

No patient or public contribution.

## Impact

The study addresses the issue of nursing professionalism in a rapidly evolving healthcare landscape, emphasizing its crucial role in ensuring patient safety and quality care.

More than half of the nurses reported having high levels of professionalism. Professional qualifications, up-to-date training, and career opportunities were identified as key factors associated with high nursing professionalism.

The findings can serve as a foundation for developing policies and programs aimed at improving professionalism among nurses, with potential implications for patient outcomes.

The emphasis on the significance of professional qualifications and up-to-date training suggests the need for continuous education and training programs to enhance nursing professionalism.

## Introduction

In the rapidly evolving healthcare landscape [1], professionalism among nurses plays a pivotal role in ensuring patient safety and quality care. The nursing profession has undergone a transformation, especially since the onset of the pandemic, evolving from a job into a profession marked by precision and professional independence.

Nursing, as a profession, is characterized by a set of dynamic values, dedication, obedience, commitment to societal betterment, unwavering ethical values, and a strong sense of accountability and responsibility [2, 3]. It involves delivering competent, safe, and ethical care while collaborating with clients, families, communities, and healthcare teams.

Professionalism in nursing is guided by a multifaceted set of values that forms the foundation for nurses’ knowledge and practice [4]. This professionalism extends beyond technical competence and is rooted in ethical decision-making and adherence to practice guidelines and standards [5, 6].

Few studies have revealed lacunae in applying the code of ethics in nursing practice among nurses and nursing students [7, 8]. Furthermore, a systemic review has indicated that a poorly perceived nursing profession can lead to poor patient outcomes [9]. Several studies have revealed a gap between personal and professional values among nursing professionals [10, 11], emphasizing the need to integrate professional values into nursing education. These discrepancies can significantly impact patient outcomes and influence nurses’ intention to leave the profession [12].

Thus, there is an urgent need to assess the level of professionalism and its associated factors among nurses. In India, until recently, only a few studies have explored nurses’ perspectives on professionalism. Therefore, we aimed to assess the level of professionalism and explore the factors affecting professionalism among nurses in a tertiary care center, which represents a first step in developing policies and programs for nurses.

## Methods

### Study design and setting

An institutional-based descriptive cross-sectional study was conducted from October 2022 to March 2023 to measure the level of professionalism and associated factors among nurses working at a tertiary care center in Eastern India using a total enumeration sampling technique.

### Study participants

The source population for this study consisted of nurses employed in the hospital. The study population included all nurses who met the inclusion criteria and agreed to participate. The inclusion criteria consisted of nurses working in the hospital and available during data collection; those with less than 6 months of working experience were excluded from the study. We distributed a Google Form link to 288 registered nurses via WhatsApp and Gmail to complete the questionnaires. We received responses from 270 nurses, resulting in a response rate of 93.7%. The final data were collected from the pilot study.

### Ethical considerations

The Institutional Ethics Committee of the procuring institute reviewed the protocol, and permission was granted to carry out the study vide no- IEC/AIIMS/Kalyani/Meeting/2022/46 dated 22/07/2022. All participants were informed about the purpose of the study and their participation was completely voluntary. Written informed consent was obtained from all the eligible participants. The participants were also assured of the confidentiality and anonymity of the obtained information.

### Sample size

The sample size was calculated using the formula; n = Z² P (1 − P)/d². Considering the prevalence of high professionalism among nurses (P) as 68.6% [23] at a 95% confidence interval (Z = 1.96) with a 6% maximum allowable error (d). By inserting these values into the formula, we got a calculated sample size of 235. Furthermore, for a 10% nonresponse rate, the required sample size was set to 260.

### Measures

Tool I consisted of sociodemographic, personal, and organizational characteristics such as age, gender, marital status, working experience, personal and job satisfaction, effective interpersonal relationships with patients and healthcare teams, up-to-date training, career opportunities, location of the institute, and satisfaction with the work schedule.

Tool II comprises a 38-item Nurse Professionalism Scale, initially developed by Braganca et al. [28], which assesses the professional behavior of nurses while performing roles and responsibilities related to the patient care activities on a five-point Likert scale (0 = Not Applicable; 1 = Never; 2 = Rarely; 3 = Sometimes; 4 = Mostly; 5 = Always). This scale includes six domains: professional responsibility and accountability, nursing practice, communication, and interpersonal relationships, valuing human beings, management, and professional advancement with total scores ranging from 0 to 190. A score ≥ 115 indicated high professionalism, 77–114 indicated moderate professionalism and a score less than 77 indicated low professionalism. The Cronbach’s alpha for nursing professionalism in the present study was 0.97.

### Statistical analysis

The collected data were checked for completeness and accuracy before analysis and then coded and summarized in the master data sheet. All statistical analyses were performed with SPSS Software version 26.0 utilizing both descriptive and inferential statistics. For descriptive statistics, the frequency, percentage, mean, standard deviation, and range were calculated. The Kolmogorov–Smirnov test was used to evaluate the normality of the distribution of the outcome variables. Due to the nonnormal distribution, nonparametric tests (Mann–Whitney U and Kruskal–Wallis H) were used to compare means. Binary and multivariable logistic regression analyses were carried out to identify factors associated with nursing professionalism. Model fitness was assessed using the Hosmer–Lemeshow goodness-of-fit test (*p* = 0.83), which indicated a well-fitted model. Additionally, all variables satisfied the chi-square assumption, and their odds ratios were examined. To assess multicollinearity among continuous variables, variance inflation factor (VIF) values were computed and found within the acceptable range (1 to 2), confirming the absence of multicollinearity. Bivariate and multivariate logistic regression analyses were employed to identify factors associated with outcome variables. Variables with a *p-value* less than 0.2 in the bivariable analysis were included in the multivariable analysis. Significant associations with outcome variables were determined based on a *p-value* less than 0.05 with a 95% confidence interval.

## Results

The mean age of the nurses was 27.33 ± 2.75 years, with the majority belonging to 23–27 years age group (59.6%), female (82.6%), unmarried (70.4%), and having professional experience of 2 to 5 years (47.4%). Furthermore, the majority of participants reported satisfaction with their current job (67.8%), the location of the institute (66.7%), and work schedule (80.7%). (Table 1)

**Table 1:** Demographic, personal and organizational characteristics of nurses (*N* = 270)

Demographic characteristics	Frequency (%)
Age (years) Mean ± SD (Range)	27.33 ± 2.75 (23–36)
Age group (years) Young [17] (23-27) Middle (28–32) Old (33–36)	161 (59.6) 96 (35.6) 13 (4.8)
Gender Male Female	47 (17.4) 223 (82.6)
Marital status Unmarried Married	190 (70.4) 80 (29.6)
If married, having child No Yes	223 (82.6) 47 (17.4)
Professional qualification Diploma in Nursing BSc Nursing MSc Nursing	30 (11.1) 211 (78.2) 29 (10.7)
Total working experience (years) < 1 2–5 > 5	74 (27.4) 128 (47.4) 68 (25.2)
**Personal characteristics**	
Personal satisfaction Satisfied Not satisfied	109 (40.4) 161 (59.6)
Job satisfaction Satisfied Not satisfied	87 (32.2) 183 (67.8)
Effective interpersonal relationship with patients Yes No	50 (18.5) 220 (81.5)
Effective interpersonal relationship with healthcare team Yes No	119 (44.1) 151 (55.9)
Up-to-date training Yes No Up to some extent	146 (54.1) 30 (11.1) 94 (34.8)
Career Opportunity Adequate Not adequate	174 (64.4) 96 (35.6)
Participating in the research projects Yes No	141 (52.2) 129 (47.8)
**Organizational characteristics**	
Location of institute Satisfactory Unsatisfactory	180 (66.7) 90 (33.3)
Work Schedule Satisfied Not satisfied	218 (80.7) 52 (19.3)
Human Resource availability Adequate Not adequate	212 (78.5) 58 (21.5)
Material Resource availability Adequate Not adequate	144 (53.3) 126 (46.7)
Colleagues performance appraisal Yes No	239 (88.5) 31 (11.5)

*Abbreviations* B.Sc. Nursing-Bachelor of Science in Nursing; M.Sc. Nursing- Master of Science in Nursing

Table 2 shows the level of nursing professionalism among the participants. More than half of the nurse participants exhibited high nursing professionalism (53%), while approximately one quarter had moderate (23.0%) or low nursing professionalism (24.0%).

**Table 2:** Level of Nursing Professionalism among Nurses (*N* = 270)

Variable	Frequency (%)	95% CI	Skew	Kurtosis	Z (p)
Nursing professionalism level High Moderate Low	143 (53.0) 62 (23.0) 65 (24.0)	142.08-146.17 89.98–95.89 57.79–62.65	− 0.306	-1.348	0.156 (< 0.001)

*Abbreviation* CI- Confidence Interval

The total median score (Q1-Q3) for professionalism among nurses was 120.50 (77.7–146.0). The highest median score was observed in the area of professional responsibility (29.0), followed by management (28.0), while relatively lower scores were observed in the domains of communication and interpersonal relationships (13.0) and valuing human beings (13.0). (Table 3)

**Table 3:** Subscale of Nursing Professionalism among Nurses (*N* = 270)

Subscale of Nursing Professionalism	Mean ± SD	Median (Q1-Q3)
Professional responsibility	25.76 ± 10.39	29.00 (15.75-34.00)
Nursing practice	20.93 ± 8.17	23.00 (14.00–17.00)
Communication and Interpersonal Relationships	12.36 ± 5.02	13.00 (8.00–16.00)
Valuing Human Beings	12.22 ± 4.64	13.00 (8.00–16.00)
Management	26.21 ± 9.45	28.00 (18.00–34.00)
Professional Advancements	14.56 ± 5.67	16.00 (10.00–19.00)
Total	112.17 ± 37.61	120.50 (77.75–146.00)

Table 4 shows the mean differences in nursing professionalism according to sociodemographic, personal, and organizational variables. There was a significant difference in the mean rank between professional qualification, job satisfaction, and career opportunity and nursing professionalism scores.

**Table 4:** Mean difference in Nursing Professionalism according to demographic, personal and organizational variables

Variables	Mean ± SD	Mean Rank	U/H (p)
Age group Young (23-27) Middle (28-32) Old (33-36)	109.79 ± 37.59 116.58 ± 38.23 109.08 ± 32.60	130.51 144.81 128.54	2.125 (0.345)
Gender Male Female	112.85 ± 35.92 112.03 ± 38.04	135.26 135.55	5229.0 (0.981)
Marital status Unmarried Married	110.65 ± 38.46 115.79 ± 35.50	132.91 141.64	7108.5 (0.401)
If married, having child No Yes	110.77 ± 38.12 118.83 ± 34.69	132.65 149.02	4605.0 (0.191)
Professional qualification Diploma in Nursing BSc Nursing MSc Nursing	95.73 ± 36.52 112.85 ± 37.24 124.21 ± 36.95	99.78 137.21 160.00	9.234 (0.010)*
Total working experience (years) < 1 2–5 > 5	116.30 ± 36.93 108.79 ± 38.63 114.04 ± 36.34	144.57 127.41 148.85	2.693 (0.260)
Personal satisfaction Satisfied Not satisfied	116.78 ± 35.30 109.05 ± 38.90	145.46 128.76	7689.0 (0.085)
Job satisfaction Satisfied Not satisfied	118.70 ± 36.14 109.07 ± 38.00	150.76 128.76	6632.5 (0.027)*
Effective interpersonal relationship with patients Yes No	112.80 ± 36.32 112.03 ± 37.98	137.62 135.02	5394.0 (0.832)
Effective interpersonal relationship with healthcare team Yes No	111.03 ± 39.00 113.07 ± 36.58	134.29 136.45	8841.0 (0.822)
Up-to-date training Yes No Up to some extent	109.01 ± 35.67 108.37 ± 43.71 118.30 ± 38.13	128.08 129.67 148.89	4.250 (0.119)
Career Opportunity Adequate Not adequate	128.45 ± 30.85 82.66 ± 30.12	167.37 77.74	2807.0 (< 0.001)*
Participating in the research projects Yes No	113.43 ± 35.70 110.79 ± 39.69	136.82 134.05	8908.0 (0.771)
Location of institute Satisfactory Unsatisfactory	110.43 ± 38.58 115.66 ± 35.54	132.50 141.51	7559.5 (0.371)
Work Schedule Satisfied Not satisfied	113.14 ± 38.02 108.10 ± 35.93	137.44 127.36	S5244.5 (0.403)
Human Resource availability Adequate Not adequate	111.19 ± 37.72 108.13 ± 38.63	134.14 140.48	5859.5 (0.583)
Material Resource availability Adequate Not adequate	115.70 ± 36.47 108.13 ± 38.63	141.97 128.11	8140.5 (0.146)
Colleagues performance appraisal Yes No	112.69 ± 37.12 108.19 ± 41.67	136.36 128.89	3499.5 (0.616)

*Abbreviations* B.Sc. Nursing-bachelor of science in nursing; M.Sc. Nursing- master of science in nursing

Table 5 depicts the factors associated with nursing professionalism for the study variables. According to our multivariable regression analysis, three variables, professional qualification, up-to-date training, and career opportunity, demonstrated significant associations with high nursing professionalism. Similarly, compared with those with a diploma, nurses with a graduate nursing qualification had 4.77 times greater odds of having high nursing professionalism (AOR = 4.77, 95% CI = 1.16–19.68). Furthermore, nurses who had received up-to-date training had 4.13 times greater odds of having high professionalism (AOR = 4.13, 95% CI = 1.88–9.06), while those with adequate career opportunities exhibited substantial 33.91 times greater odds of having high nursing professionalism (AOR = 33.91, 95% CI = 14.48–79.39) than did their counterparts.

**Table 5:** Factors Associated with Nursing Professionalism among Nurses

Variables	High Professionalism Frequency (%)		COR (95% CI)	AOR (95% CI)
	Yes (*N* = 143; 53.0%)	No (*N* = 127; 47.0%)		
Age group Young (23-27) Middle (28-32) Old (33-36)	80 (49.7) 57 (59.4) 6 (46.2)	81 (50.3) 39 (40.6) 7 (53.8)	0.86 (0.27–2.69) 0.58 (0.18–1.87) 1	
Gender Male Female	24 (51.1) 119 (53.4)	23 (48.9) 104 (46.6)	1.097 (0.58–2.05) 1	
Marital status Unmarried Married	97 (51.1) 46 (57.5)	93 (48.9) 34 (42.5)	1.29 (0.76–2.19) 1	
If married, having child No Yes	116 (52.0) 27 (57.4)	107 (48.0) 20 (42.6)	1.24 (0.66–2.35) 1	
Professional qualification Diploma in Nursing BSc Nursing MSc Nursing	10 (33.3) 112 (53.1) 21 (72.4)	20 (66.7) 99 (46.9) 8 (27.6)	1 5.25 (1.72–15.98)* 2.32 (0.98–5.47)	1 4.77 (1.16–19.68)* 2.04 (0.67–6.17)
Total working experience (years) < 1 2–5 > 5	42 (56.8) 67 (52.3) 34 (50.0)	32 (43.2) 61 (47.7) 34 (50.0)	0.76 (0.39–1.47) 0.91 (0.50–1.64) 1	
Personal satisfaction Satisfied Not satisfied	62 (56.9) 81 (50.3)	47 (43.1) 80 (49.7)	1.30 (0.79–2.12) 1	
Job satisfaction Satisfied Not satisfied	51 (58.6) 92 (50.3)	36 (41.4) 91 (49.7)	1.40 (0.83–2.34) 1	
Effective interpersonal relationship with patients Yes No	25 (50.0) 118 (53.6)	25 (50.0) 102 (46.4)	0.86 (0.46–1.59) 1	
Effective interpersonal relationship with healthcare team Yes No	61 (51.3) 82 (54.3)	58 (48.7) 69 (45.7)	0.88 (0.54–1.43) 1	
Up-to-date training Yes No Up to some extent	15 (50.0) 69 (47.3) 59 (62.8)	15 (50.0) 77 (52.7) 35 (37.2)	1.88 (0.73–3.86) 1 1.88 (1.10–3.19)*	0.70 (0.21–2.27) 1 4.13 (1.88–9.06)**
Career Opportunity Adequate Not adequate	131 (75.3) 12 (12.5)	43 (24.7) 84 (87.5)	21.32 (10.63–42.77)** 1	33.91 (14.48–79.39)** 1
Participating in the research projects Yes No	75 (53.2) 68 (52.7)	66 (46.8) 61 47.3)	1.01(0.63–1.64) 1	
Location of institute Satisfactory Unsatisfactory	91 (50.6) 52 (57.8)	89 (49.4) 38 (42.2)	0.74 (0.44–1.24) 1	
Work Schedule Satisfied Not satisfied	119 (54.9) 24 (46.4)	99 (45.4) 28 (53.8)	1.40 (0.76–2.57) 1	
Human Resource availability Adequate Not adequate	108 (50.9) 35 (60.3)	104 (49.1) 23 (39.7)	0.68 (0.37–1.23) 1	
Material Resource availability Adequate Not adequate	82 (56.9) 61 (48.4)	62 (43.1) 65 (61.6)	1.40 (0.87–2.27) 1	1.93 (0.99–3.76) 1
Colleagues performance appraisal Yes No	128 (53.6) 15 (48.4)	111 (46.4) 16 (51.6)	1.23 (0.58–2.60) 1	

*Abbreviations* AOR, adjusted odds ratio; COR, crude odds ratio; B.Sc. Nursing-Bachelor of Science in Nursing; M.Sc. Nursing- Master of Science in Nursing

Highly significant = **p-value < 0.01, *p-value ≤ 0.05

## Discussion

This study focused on the level of professionalism among nurses and associated factors among 270 nurses working in a tertiary-level hospital in India. The results showed that more than half of the nurse participants exhibited high levels of professionalism, with variations across different domains. Professional qualifications, up-to-date training, and career opportunities were identified as key factors associated with high professionalism among nurses.

Nursing professionalism is a global concern, with variations observed across different countries and healthcare systems. The findings of our study revealed that more than half of the nurses had high professionalism, which was in line with the findings of various studies conducted in Ethiopia [6, 21]. This high level of professionalism in our study participants can be attributed to younger age, adequate staffing ratio, resource availability, job security, and support for professional development opportunities in our institute, which could impact nurses’ motivation, job satisfaction, and, consequently professionalism [22].

Nursing professionalism is multidimensional, dynamic, and culture-oriented [5]. It may be influenced by various organizational, educational, and societal factors, which can vary significantly across countries. Studies among Japanese and Ethiopian nurses reported low levels of professionalism among nurses [23, 24]. Another study showed professionalism as a common factor influencing job satisfaction in Korean and Chinese nurses [25]. Iranian nurses’ attitude towards professionalism was reported to be at an average level [26].

In this study, the highest median scores were attributed to the subdomain of professional responsibility and management. In contrast, another study attributed high scores to subdomains such as ‘maintaining the confidentiality of the patient’ and ‘safeguarding the patient’s right to privacy’ [2]. A Korean study depicted that higher professionalism among oncology nurses may lead to higher compassion satisfaction and lower compassion fatigue [18].

One of the interesting findings of our study is that nurses with more than 5 years of experience had higher mean scores on professionalism, which is supported by various studies that revealed high professionalism among highly experienced nurses [19, 26]. On the other hand, a recent survey in India indicated that nurses with fewer years of experience exhibited greater professional values compared to their more experienced counterparts [3]. 

Professionalism in nursing practice is important for ensuring patient safety, quality care, and positive healthcare outcomes. Another intriguing finding of this study is that participants who are personally and job-satisfied and have effective interpersonal relationships with patients attain higher professional median scores. These finding aligns with those of other studies indicating that nurses who are satisfied with their peers have greater job satisfaction and, consequently greater professional value [11, 20].

Multivariate regression analysis revealed that professional qualifications, up-to-date training, and career opportunities were significantly associated with nursing professionalism. A previous study showed that nurses with a diploma qualification exhibited high professionalism scores [19]. This finding contrasts with the present study, which demonstrated that nurses with a graduate degree exhibited high levels of professionalism. Other studies have noted that age, number of years of experience, and length of service significantly contribute to the nursing profession [6].

Continuous education plays a significant role in making learning more concrete, helping in pouring professional values and fostering deeper commitment to the profession [13]. Another major finding of this study is that nurses who have undergone up-to-date training exhibit higher levels of nursing professionalism, which has been supported by several studies [2, 14, 15]. These findings may be attributed to the continuous enrichment of knowledge and values through participation in conferences and workshops after graduation [16].

In our study, we did not find any influence of gender on nursing professionalism. In contrast, a study reported that female nurses had high professionalism [23]. This discrepancy could be due to differences in participant characteristics, such as professional qualification, age, educational attainment, location, and study period [17]. A recent study illustrated that nursing professionalism plays a mediating role in the relationship between self-efficacy and job embeddedness [27]. 

This comprehensive study provides valuable insights into the factors influencing nursing professionalism, covering various dimensions, such as sociodemographic, personal, and organizational factors. Additionally, we used validated tools for data collection and managed to acquire an adequate sample size with high response rates.

### Limitations of the study

Our study has several limitations. The cross-sectional study design and single time point data do not allow for the examination of changes or trends over time. In addition, self-report bias may be introduced due to self-administered questionnaires and convenience sampling may introduce selection bias.

### Clinical practice relevance

Our findings have important implications for redefining the roles of nurses in India to be more in line with those in Western countries. In Western countries, individuals are prioritized for continuous education and training, and nurses often have higher educational qualifications, and clear career paths with opportunities for specialization and advancement, which are associated with greater professionalism. Redefining the roles of nurses in India might involve establishing and promoting up-to-date training programs and encouraging the pursuit of advanced degrees. Policies in India could include support for attending conferences, workshops, and international collaborations, practices common in Western countries. Such alignment may improve patient care standards, increase professional satisfaction among nurses, and enhance healthcare outcomes in India.

## Conclusions

To conclude, more than half of the nurse participants displayed high professionalism, particularly in domains related to professional responsibility and management. Factors associated with nursing professionalism include professional qualifications, up-to-date training, and career opportunities.

## Data Availability

The data that support the findings of this study are available on request from the corresponding author. The data are not publicly available due to privacy or ethical restrictions.
